# Low platelet count as risk factor for infections in patients with primary immune thrombocytopenia: a retrospective evaluation

**DOI:** 10.1007/s00277-018-3367-9

**Published:** 2018-05-18

**Authors:** Mingming Qu, Qiang Liu, Hong-Guo Zhao, Jun Peng, Heyu Ni, Ming Hou, A. J. Gerard Jansen

**Affiliations:** 1 Department of Hematology, Qilu Hospital, Shandong University, 107 Wenhuaxi Road, Jinan, China; 2 Key Laboratory of Cardiovascular Remodeling and Function Research, Chinese Ministry of Education and Chinese Ministry of Health, Jinan, China; 3 Department of Hematology, The Affiliated Hospital of Qingdao University Medical College, Qingdao, China; 4 Toronto Platelet Immunobiology Group, Toronto, ON Canada; 5 Department of Laboratory Medicine, Keenan Research Centre for Biomedical Science of St Michael’s Hospital, Toronto, ON Canada; 6 Canadian Blood Services, Ottawa, ON Canada; 7 Department of Laboratory Medicine and Pathobiology, University of Toronto, Toronto, ON Canada; 8 Department of Hematology, Erasmus Medical Center Rotterdam, Erasmus MC Cancer Institute, Groene Hilledijk 301, 3075 EA Rotterdam, The Netherlands; 9 Department of Plasma Proteins, Sanquin-AMC Landsteiner Laboratory, Amsterdam, The Netherlands

**Keywords:** ITP, Infection, Platelet transfusion, Thrombocytopenia

## Abstract

Infectious complications are common and sometimes life threatening in patients with immune thrombocytopenia (ITP), mainly due to the immune-suppressive therapy. Recent evidence suggests a potential role of platelets in the inflammation process. In this clinical study, we further investigated the role of thrombocytopenia on infections in patients with primary ITP. We retrospectively evaluated data from the recently published large randomized clinical trial of a cohort of 195 patients with primary ITP, who were randomized for prednisone or high-dose dexamethasone. From 158 patients (81%), data on platelet count and infections within the first month of treatment were collected. In this period, 24% of the ITP patients had an infection. Patients with infection had significant lower platelet counts during the first month of treatment leading to a significant lower therapy response at 1 month and a significant longer hospital stay (14.0 versus 9.8 days). Additionally, Cox regression analysis showed that an increase in platelet count of 20 × 10^9^/L led to a reduction of 52% in infections in the next week, showing low platelet count is a significant risk factor for infection. Platelet transfusion led to an increase in platelet count in ITP patients without infection, but not in patients with infection. In conclusion, infections are common in patients with primary ITP leading to significant worse response rates and a longer hospital stay. Interestingly, low platelet count was independently correlated with an increased risk of infection.

## Introduction

Immune thrombocytopenia (ITP) is an autoimmune disease characterized by autoantibody-induced platelet destruction and decreased platelet production often resulting in mild bleeding symptoms (e.g., petechiae) to severe bleeding manifestations (e.g., intracranial hemorrhage) [1, 2]. Diagnosis is made by exclusion of other causes of the thrombocytopenia, e.g., infections. In patients with chronic ITP, infections are common, with major morbidity and mortality [3, 4]. It is mainly considered that treatment of ITP increases the risk of infections (e.g., corticosteroids, and other immunosuppressive agents). However, recently, it is shown that infection is not only related to the immunosuppressive treatment in chronic ITP but also to the etiology of the disease itself [5]. New studies show that platelets play a role in the immune response, but knowledge of the clinical importance is still limited, especially in primary ITP patients [6–10]. To further elucidate the role of platelets in risk of infection, we analyzed data of a cohort of primary ITP patients from the recently published prospective randomized multicenter clinical trial of Wei et al. [11].

## Materials and methods

### Study design

In the prospective, multicenter, randomized, controlled, open-label clinical trial of Wei et al., newly diagnosed ITP patients were randomized for high-dose dexamethasone (HD-DXM) or prednisone (PDN) for first-line management [11]. Patients in the HD-DXM arm received DXM orally at 40 mg for 4 consecutive days and then stopped. If platelet count remained low (< 30 × 10^9^/L) or there were bleeding symptoms by day 10, an additional 4-day course of DXM (40 mg daily) was given. Patients in the PDN arm received PDN orally at 1.0 mg/kg body weight daily for four consecutive weeks. No dose adjustments were made so all patients in both groups received the intended and same dose of immunosuppressive therapy. Platelet count was measured according routine local laboratory practice at days 0, 7, 14, 21, and 28 after randomization. The study was conducted in collaboration among nine separate investigation sites in China. Data were collected from each participating site and sent to the principal investigation site at Qilu Hospital, Shandong University, for analysis. The study protocol was approved by the ethics committee on medical research of each participating site. All patients provided written informed consent in accordance with the Declaration of Helsinki before enrollment.

### Patients

Inclusion criteria were patients with primary ITP, without prior treatment. Exclusion criteria were seropositive detection of HIV, hepatitis B virus or hepatitis C virus, pregnancy or lactation, active infection, hypertension, diabetes, cardiovascular diseases, liver and kidney function impairment, psychosis and osteoporosis, and life-threatening bleeding (e.g., massive hemorrhage with severe anemia, central nervous system bleeding). No prophylactic antibiotics were given during first month of treatment with steroids.

### Infections

Primary outcome in the study of Wei et al. was initial response and sustained response based on platelet counts. As adverse event, three infections were measured in this study [11]. Definition of infection was different than ours; they measured only severe infections caused by immunosuppressive therapy (e.g., one patient terminated immunosuppressive therapy due to infection). As our focus is on the role of platelets on infection, we used a different, broader, definition for infections. In our study, infections with a grade 2 or more were scored according to the Common Terminology Criteria for Adverse Events (CTCAE) grading scale (version 4.03: June 14, 2010), including viral, bacterial, and fungal episodes.

### Statistical analysis

Baseline characteristics were summarized using descriptive statistics. Continuous variables were summarized as median or mean ± standard deviation or standard error of the mean, and categorical variables were summarized using frequencies and percentages. Differences between the groups were analyzed with *t* tests or with nonparametric Mann-Whitney test when not normally distributed. To evaluate the relationship between platelet count and infections, we used Spearman correlation coefficient. To adjust for time of infection, we used Cox regression analysis with platelet count as time-varying covariate; platelet counts were categorized into groups of 20 × 10^9^/L, and the last platelet count before the time of occurrence of infection was used for analysis, e.g., when infection occurred at 10 days after randomization, the platelet count from day 7 was used. Risk of infection was presented as hazard ratio. All *p* values are two-sided, and *p* values < 0.05 were considered statistically significant.

## Results

### Baseline characteristics

Between January 2011 and May 2014, 261 patients were screened for eligibility and 195 patients with primary ITP were randomized in the study of Wei et al. [11]. From this patient population, data on platelet count and infection of 158 patients could be collected retrospectively. In the first month after randomization, infection occurred in 24% of the patients: 120 patients had no infection and 38 patients had a viral or bacterial infection. Baseline characteristics of both patient groups are summarized in Table 1. There were no differences in age, sex, antibodies, and type of treatment. Except for leucocyte count at *t* = 0, no differences were found for blood values between the two study groups. Usage of type of steroids was similar for both groups.

**Table 1:** Patient demographics and baseline characteristics

Characteristic	Overall (*N* = 158)	No Infections (*N* = 120)	Infections (*N* = 38)
Age (year)	42 ± 16	42 ± 16	42 ± 16
Sex (m/f)	51/107	37/83	14/24
Hemoglobin value (mg/dL) at *t* = 0	123.2 ± 28.6	122.1 ± 30.8	126.8 ± 20.1
Leucocyte count (× 10^9^/L) at *t* = 0	7.7 ± 3.4	7.2 ± 3.2	9.1 ± 3.6*
Platelet count (× 10^9^/L) at *t* = 0	13.4 ± 12.2	13.8 ± 12.8	11.8 ± 10.4
Hemoglobin value (mg/dL) at *t* = 1 month	125.5 ± 32.2	124.0 ± 23.9	130.4 ± 50.3
Leucocyte count (× 10^9^/L) at t = 1 month	11.0 ± 7.9	11.2 ± 7.8	10.5 ± 8.2
Platelet count (× 10^9^/L) at *t* = 1 month	87.7 ± 78.7	107.4 ± 79.7	25.4 ± 24.3*
Bleeding score	3.9 ± 3.4	3.8 ± 3.5	4.1 ± 3,2
Duration hospital stay (days)	10.9 ± 6.5	9.8 ± 5.2	14.0 ± 8.7*
Treatment (dexamethasone/prednisone)	118/40	87/33	31/7
Antibodies			
None	46	35	11
GPIb-IX	13	11	2
GPIIbIIIa	15	7	8
Both	18	16	2
Unknown	66	51	15
Type of infection			
Viral			11
Bacterial			27
Time of infection (day)			5.7 ± 5.1
Platelet transfusion			
Number of patients transfused	91 (57.6%)	62 (51.7%)	29 (76.3%)
Total amount of units	150	91	59
Median amount of units	1	1	1
Mean	1.0	0.8	1.6*

Mean values for age, platelet count, leucocyte count, hemoglobin values, bleeding score, hospital stay, and time of infection ± standard deviations are shown. Bleeding score was calculated by adding the points relevant to various clinical bleeding signs as described by Wei et al. [11]

*p < 0.01

### Infection leads to lower response rates and prolonged hospital stay

**Fig. 1:**
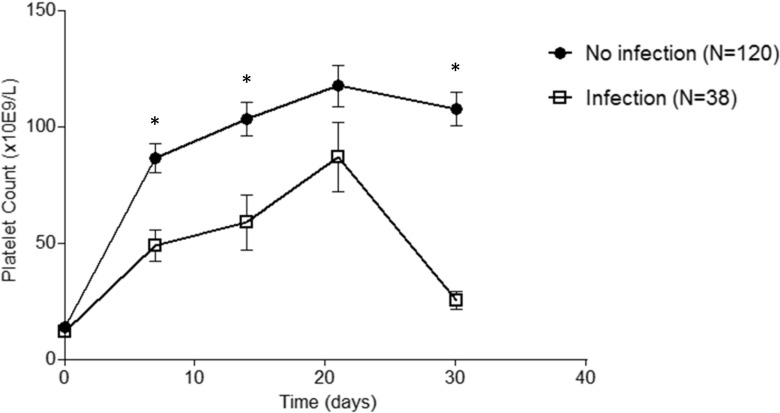
Infection leads to lower platelet counts in patients with primary ITP. Mean platelet counts (± s.e.m.) during the first month after therapy in patients with primary ITP with and without infection. *p < 0.01

**Fig. 2:**
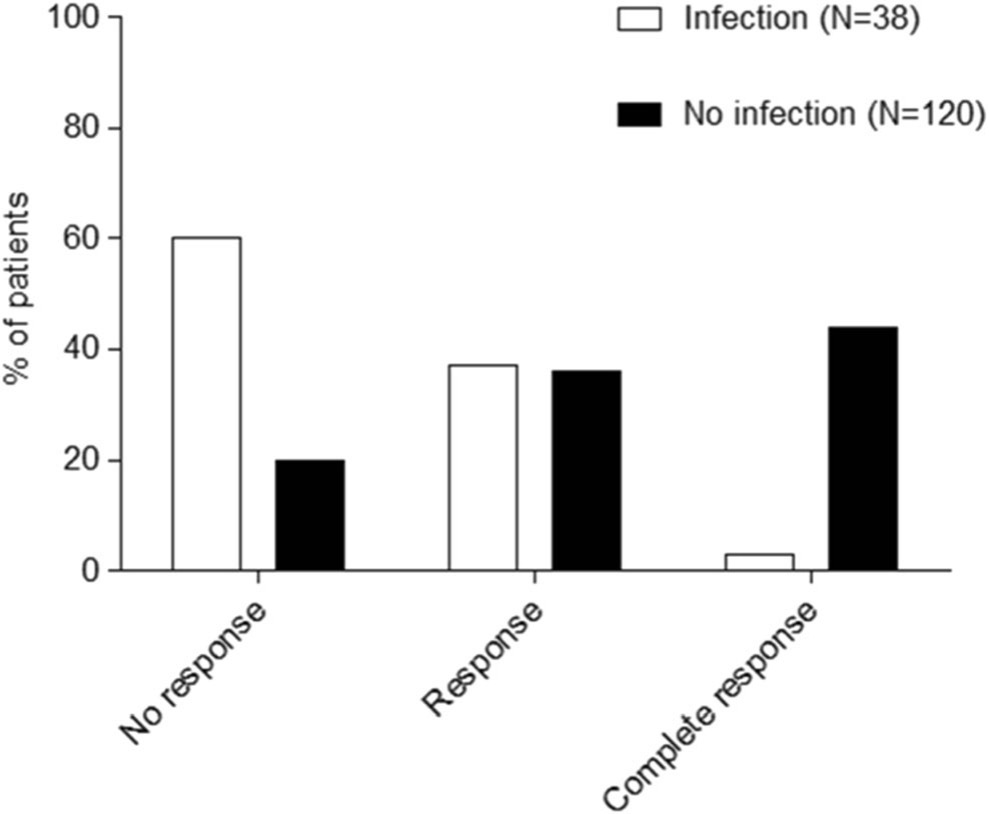
Infection leads to lower response rates at 1 month. Response according to Rodeghiero et al. (No response: platelet count < 30 × 10^9^/L; response: platelet count > 30 × 10^9^/L and < 100 × 10^9^/L); complete response: platelet count > 100 × 10^9^/L [2]. *p* < 0.05

Mean platelet counts during the first month after randomization are shown for patients with and without infection in Fig. 1. Platelet count at t = 0 was the same for both groups: 13.8 × 10^9^/L for the patients without infection and 11.8 × 10^9^/L for the patients with infection (*p* > 0.05) (Table 1). However, during the first month, patients with infection had significant lower platelet counts than patients without infections at 1, 2, and 4 weeks (*p* < 0.01). Only, the lower platelet count at 3 weeks for the patients with infection was not statistically significant. According to the response criteria of Rodeghiero et al., a significant lower response at 1 month is found for patients with infection as shown in Fig. 2 (*p* < 0.05) [2]. Additionally, infection is also associated with a significant longer hospital stay, e.g., 9.8 ± 5.2 days for patients without infection and 14.0 ± 8.7 days for patients with infection (Table 1) (*p* < 0.01). In both groups, none of the patients had a thrombotic event, e.g., deep venous thrombosis or pulmonary embolism, during the first month of treatment. At 1 month, no differences were found between patients with and without infection in mean hemoglobin value (124.0 vs 130.4 g/L, *p* > 0.05) and leucocyte count (11.2 × 10^9^ vs 10.5 × 10^9^/L, *p* > 0.05).

### Low platelet count is correlated with increased risk of infection

As shown in Fig. 1, lower platelet counts led to more infections. Platelet count at 1 month was significantly correlated with infection with a Spearman correlation coefficient of − 0.517 (*p* < 0.01). To further adjust for time of infection, we used Cox regression analysis with platelet count as time-dependent variable. Platelets were categorized into groups of 20 × 10^9^/L, and the last platelet count before time of infection was used for analysis as was described in the “Materials and methods” section. A hazard ration of 0.52 was found meaning an increase in platelet count of 20 × 10^9^/L leads to a significant reduction of 52% in chance of infection in the next week (HR 0.52, 95% CI 0.35–0.77, *p* = 0.001) (Table 2). Adding leucocyte count at *t* = 0, which was significant (Table 1), to the model platelet count remained a significant risk factor for infection (Table 2).

**Table 2:** Platelet count is a risk factor for infection

	Hazard ratio	Standard error	95% CI	*P* value
Platelet count	0.52	0.10	0.35–0.77	0.001
Platelet count Leucocyte count	0.54 1.10	0.11 0.04	0.37–0.79 1.01–1.19	0.002 0.021

Last platelet count before occurrence of infection was used. Platelets were grouped into groups per 20 × 10^9^/L. Cox regression analysis with platelet count as time-dependent variable was used. Secondary analysis was performed adding leucocyte count to the model

### Platelet transfusion in ITP patients with and without infection

**Fig. 3:**
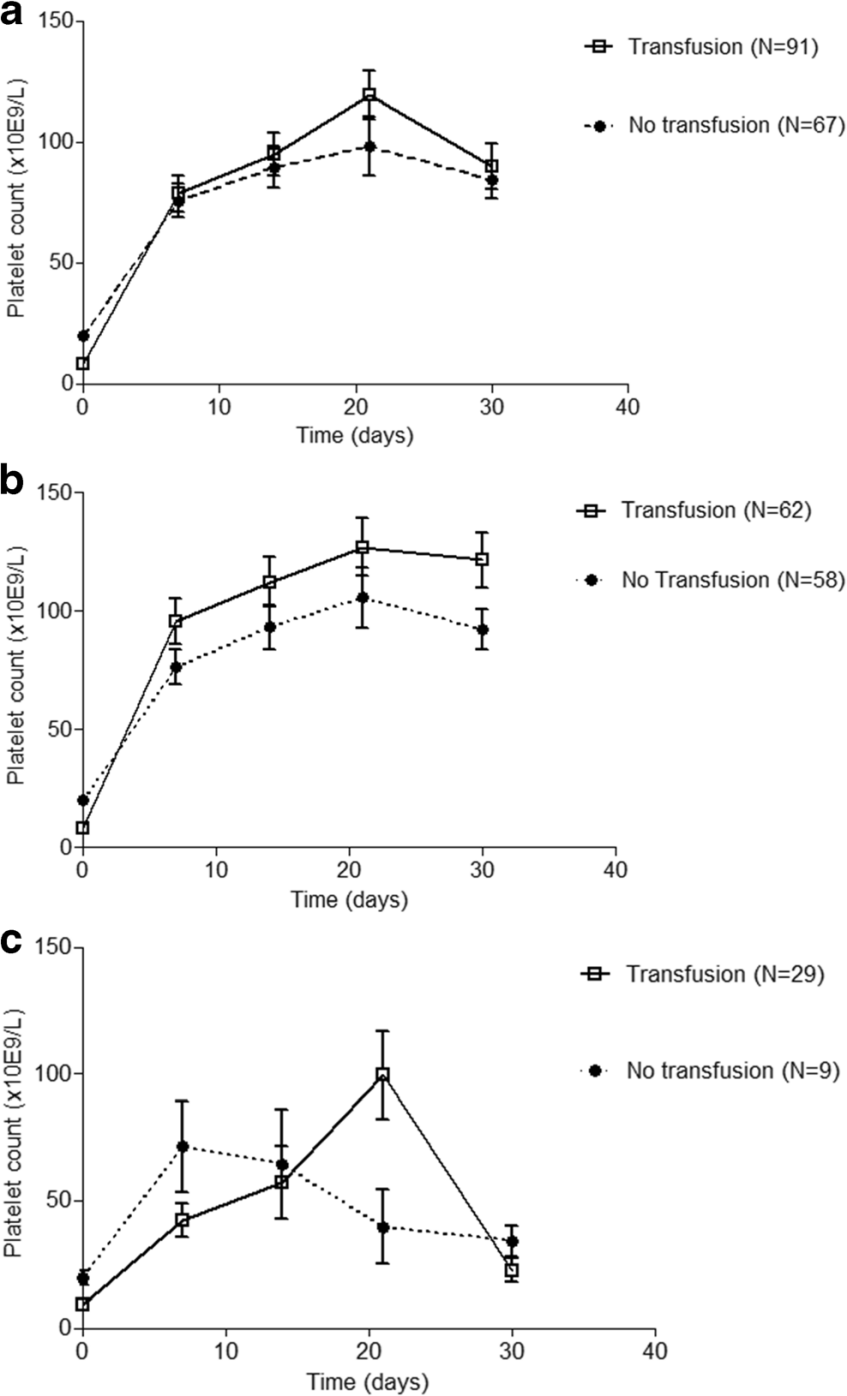
**a** Platelet transfusion does not increase platelet count in patients with ITP. Median platelet counts are shown per patient group. Platelet count at *t* = 0 was significantly lower in patients who received platelet transfusion (*p* < 0.01). No significant differences were seen at 1, 2, 3, and 4 weeks. **b** Platelet transfusion increases platelet count in primary ITP patients without infection. Platelet transfusion leads to higher platelet counts from day 7 in patients with primary ITP without infection. Median platelet counts are shown for both groups. No significant differences were found. **c** Platelet transfusion does not increase platelet count in primary ITP patients with infection. In patients with primary ITP with infection, there is no effect of platelet transfusion on platelet count. Median platelet counts are shown for both groups. No significant differences were found

Ninety-one patients (58%) received one or more platelet transfusions during the first month of treatment, where 85% of the units were transfused within the first week (mean 4.6 ± 4.2 days after inclusion) (Table 1). At *t* = 0, there is a significant lower platelet count in the patient group who received a transfusion: 8.5 × 10^9^/L versus 19.9 × 10^9^/L (*p* < 0.01) (Fig. 3a). During the first month, there is no effect of platelet transfusion on platelet count (*p* > 0.05) (Fig. 3a). In the patients without infection, there is a positive, but not significant, trend of platelet transfusion on platelet count (Fig. 3b). In the patients with infection, there seems no effect of platelet transfusion on platelet count (Fig. 3c).

## Discussion

The results of this large cohort reveals that an incidence of 24% in the first month of treatment infection is a common problem in patients with primary ITP. Additionally, patients with infection had a significant lower response rate at 1 month compared to patients without infection, possibly contributing to the significant longer hospital stay. Also, we could show that platelet count is correlated with infections and additionally that a low platelet count is an independent risk factor for infection within the next week with a hazard ration of 0.52. The role of platelet transfusion however remains unclear.

In the last years, more and more research has shown that platelets can modulate the innate and adaptive immune response. Especially for patients with sepsis, it is shown that thrombocytopenia is a surrogate marker for poor prognosis [6]. For patients with influenza infection, we could recently show that higher viral load leads to lower platelet counts and treatment with a sialidase inhibitor oseltamivir leads to higher platelet counts [12]. In the current study, we could show that infection is associated with lower response rates and longer hospital stay in patients with primary ITP. This might be due to the immunomodulatory treatment (e.g., steroids) patients received, although the average time of infection was 5.7 days after start of steroid therapy, making the role of treatment smaller in our study. Also, no difference in infection rate for dexamethasone or prednisone treatment was found. However, platelets can limit bacterial growth, influence leucocyte recruitment and functions, influence cytokine response, and influence activation of the vascular endothelium and coagulation system [8, 13–22]. Although the mechanism of these interactions is not fully understood, the expression of functional CD154 (CD40L) is a main contributor [23]. Interestingly, we could show that thrombocytopenia itself may be an independent and significant risk factor for infection, strengthening the role of platelets in infections.

Treatment of infections include antibiotic, antiviral, or antifungal therapies. To prevent infection-associated morbidity and mortality increase in platelet count, e.g., by prophylactic platelet, transfusions might be the next step. In animal models, reinfusion of platelets to 10–15% of normal platelet count did prevent lung and brain bleeding during sepsis. As in theory, platelet transfusions can be given to prevent infections; during active infection, post-transfusion increments appear lower and more platelet transfusions are needed to maintain minimal numbers [24]. The beneficial effects of prophylactic platelet transfusion is uncertain and might be influenced by the state of the infection-induced response system [25]. Specifically in patients with ITP, prophylactic platelet transfusion is unclear and therefore however is not recommended, where in emergency cases, platelet transfusions can be given in combination with intravenous immunoglobulins (IVIg) [4, 26]. Our study is the first study providing data on platelet transfusion in ITP patients with and without infections. However, the primary indication for platelet transfusion remains still severe bleeding (risk) and prophylactic transfusion is therefore not recommended.

Acute bacterial or viral infections are known to cause ITP [5, 27]. First-line treatment of patients with ITP is steroids [2]. Next treatment options include for example splenectomy, TPO agonist, rituximab, and others. Treatment response is based on increase in platelet count to decrease risk of bleeding complications. However, according to our study, an increase in platelet count also leads to a reduction in infection. As mentioned before, platelet transfusion in patients with primary ITP is not recommended however, due to the low increments and risks of transfusion [2].

Limitations of our study are the retrospective nature of collection of data on infection as this was not the purpose of the randomized clinical trial. Secondly, infection was measured as CTC grade 2, thus with clinical symptoms. As we do not know the exact moment of inoculation, we could only use the time of clinical manifestation as outcome. Theoretically, as the thrombocytopenia occurs after the inoculation of the infection and the thrombocytopenia before the clinical presentation, thrombocytopenia could still be explained by the infection itself rather than being a risk factor. However, with the weekly intervals of platelet count measurements and the average inoculation time of the viral and bacterial infections, the relationship between low platelet count and risk of infections seems true. Additionally, there were no patients who had high platelet counts before time of infection. This weakens our Cox regression model with platelet count as time-dependent variable. Also, it is possible that the thrombocytopenia of some patients, thus the diagnosis ITP, might be a precursor sign of a hematological malignancy which have an increased risk of infections. Finally, due to the nature of our study design, we could only show a correlation and not an association between platelet count and infections. Further research is needed to confirm a possible association.

In conclusion, an incidence of 24% infection is a common complication in patient with primary immune thrombocytopenia leading to a worse response rate at 1 month and a longer hospital stay. Low platelet count is correlated with increased risk of infections, strengthening the role of platelets in inflammation, but the role of platelet transfusion as therapeutic tool remains unclear. Further research is needed to investigate the exact mechanism of platelets in the inflammation process and the opportunity as therapeutic tool.
